# Investigation of the effects of a high fish diet on inflammatory cytokines, blood pressure, and lipids in healthy older Australians

**DOI:** 10.3402/fnr.v58.20369

**Published:** 2014-01-15

**Authors:** Jessica A. Grieger, Michelle D. Miller, Lynne Cobiac

**Affiliations:** 1 Department of Nutrition and Dietetics, Flinders Clinical and Molecular Medicine, Flinders Medical Centre, Bedford Park, Australia; 2 CSIRO Preventative Health Flagship, Adelaide, Australia

**Keywords:** older adults, fish, C-reactive protein, omega-3 long chain fatty acids, blood pressure, lipids

## Abstract

**Background:**

Aging is a condition of chronic inflammation. In healthy Australians ≥64 years, the primary aim was to determine whether four servings/week of mixed fish (FISH) improves serum cytokines (i.e. C-reactive protein (CRP), IL-1, IL-6, TNF-α) compared to a diet low in fish (<1 serving/week, CONTROL); the secondary aims were to assess the effect of the diet on blood pressure and serum lipids (TC, HDL-C, TG, calculated LDL-C).

**Methods:**

An 8-week randomized, parallel study, stratified by CRP (<3 mg/L vs. ≥3 mg/L) on entry to the study. Compliance was measured using 3-day weighed food records in weeks 1 and 7 of the study. A 12-h fasting blood sample was taken at baseline and 8-weeks for erythrocyte fatty acids as confirmation of compliance, and measurement of serum cytokines and lipids. Blood pressure was measured at both time points.

**Results:**

Eighty participants completed the study (mean (SD) age: 69.6 (5.8) years). During week 1 of the study, mean ± SEM daily dietary intake of very long chain omega-3 polyunsaturated fatty acids (VLCN n-3 PUFA) in FISH vs. CONTROL was 1,676±129 mg vs. 27±5 mg (*p*<0.001). Mean (SD) gram intake of study fish and meat was 121 (45) g and 123 (78) g, for those allocated to FISH and CONTROL, respectively. Mean ± SEM percentage VLCN n-3 PUFA in erythrocytes at 8-weeks was higher in those allocated to FISH vs. CONTROL (10.2±0.2% vs. 8.2±0.3%, *p*<0.001). There was no between-group difference in CRP (*n*=80), IL-1β (*n*=33) or IL-6 (*n*=21) concentrations, blood pressure, or lipids, at 8-weeks.

**Conclusions:**

Eight weeks consumption of four servings/week fish did not affect serum cytokine concentrations, blood pressure or lipids compared to a diet low in fish. In healthy older adults with low inflammatory burden, our results do not support that short-term consumption of mixed fish has a beneficial effect on selected cardiovascular biomarkers.

With aging, the inflammatory process is aggravated. This may be a result of conditions such as cachexia (1), neurodegenerative diseases (2, 3), or depression (4). It is recognized that chronic, low-grade inflammation is associated with increased risk for cardiovascular, and a number of other, chronic diseases (5, 6). With aging, blood pressure also generally increases (7) and elevated blood pressure (>140/90) is a risk factor for cardiovascular disease (CVD), specifically stroke (8). The ability of very long chain omega-3 polyunsaturated fatty acids (VLCN n-3 PUFA) that are contained in fish to impact markers of CVD continues to be investigated; however, clinical trials assessing increased intake of fish in older adults, who are at greater risk of CVD, are inconclusive.

In Australia, consumption of fish is low (9–11). In 1995, the estimated mean intake of total VLCN n-3 PUFA in those >65 years was 219 mg/day, to which fish and seafood products contributed 48% (11). In a more recent survey of older adults in Australia (*n*=854), the mean frequency of finfish/seafood consumption was 1.7 times/week (median intake 173 g), however only 28% (*n*=242) consumed ≥500 mg/day VLCN n-3 PUFA from finfish/seafood alone (12). These intakes are lower than current Australian recommendations for fish and VLCN n-3 PUFA consumption (10).

Findings from some observational studies reveal that dietary intakes of VLCN n-3 PUFA of ≥100 mg/day in Western countries (13–15) or ≥470 mg/day in the higher fish eating country of Japan (16), were associated with lower concentrations of inflammatory cytokines including C-reactive protein (CRP), IL-6, and intercellular- and vascular adhesion molecules. It is unclear whether consumption of fish in its whole form affects inflammatory cytokines in healthy older adults. Some observational studies demonstrated that fish consumption of 100–150 g/week was associated with lower cytokine concentrations (15), particularly CRP (17, 18), and studies investigating dietary patterns have found that those which included fish were also associated with lower CRP concentrations (19, 20). Some intervention studies have assessed the impact of increased fish consumption on cytokine concentrations and cardiovascular risk markers in younger (21–24) and older populations (25–31). However, specifically in the studies among older adults, the fish intervention was either in combination with other omega-3 oils or foods (28, 31), included patients with non-active/previous cancer (32) or coronary heart disease (CHD) (30), was based on guidelines to lower cholesterol (27), was only conducted in men (25, 26) or the included fish was modified by different feeds (25, 30). The current study is therefore the first to assess a high fish diet in both healthy older men and women, without risk factors for CVD, and in conjunction with their usual diet on cytokine concentrations. Only two of the previous studies compared the fish intervention to a diet in which either 150 g/day chicken was used as a reference meal (25) or a mix of 150 g/day lean pork and chicken for 5 day/week (26). In Australia, recent surveys have suggested a decline in red meat consumption, even in the older age groups, whereas consumption of poultry has been relatively constant (33). Therefore, we chose to support the consumption of red meat, appropriate for this age group, by providing four servings/week of red meat to the CONTROL group.

Only two studies in older adults have assessed fish intake on blood pressure in which a positive association was found between a higher intake of fish (34) and higher serum levels of VLCN n-3 PUFA (35) with lower blood pressure. In combination with 8-weeks of energy restriction, consumption of salmon three times/week lowered diastolic blood pressure in young, overweight, 20–40 year olds (36); and consumption of lean fish ≥4 times/week reduced blood pressure in patients with CHD (37). No RCTs were found that specifically investigated the effects of increased fish intake on blood pressure in older adults. Consumption of a range of mixed fish within the context of a usual dietary pattern in older adults, who are prone to some low-grade inflammation and who usually consume a relatively low intake of fish would therefore provide value in understanding whether increased fish intake affects cardiovascular risk markers.

The primary aims of the current study were to determine, in a group of healthy Australian adults ≥64 years of age: whether consumption of four servings/week of mixed types of fish (FISH), improves serum cytokines (i.e. CRP, IL-1, IL-6, TNF-α) compared to a typical Australian diet that is lower in fish (<1 serving/week) and higher in red meat, CONTROL. The secondary aims were to identify whether consumption of a higher fish diet improves blood pressure and serum lipids (TC, HDL-C, TG, and calculated LDL-C) compared to a typical Australian diet that is lower in fish and higher in red meat.

## Materials and methods

### Experimental design

This study was an 8-week randomized controlled, parallel study. Participants were randomized to either FISH, in which participants were provided eight servings of mixed fish per fortnight, equivalent to ∼800 mg/day EPA + DHA, or CONTROL, in which participants consumed their usual diet as well as eight servings of red meat per fortnight (equivalent to 17 mg/day EPA and 4 mg/day DHA, Table 1). The amount of meat provided was ∼0.5 serving/day (68 g/day) and was based on the recommended 1–1.5 daily servings of ‘meat, fish, poultry, eggs, nuts and legumes’ from the Australian Guide to Healthy Eating for men and women >60 years (38). Participants in both groups were advised to follow their otherwise usual dietary and physical activity patterns throughout the 8-week study. Between study groups, the food provided was designed to be no greater than 10% different for energy (kJ), protein (g), saturated fat (g) and sodium (g), except for total fat, which was nearly double in the FISH diet vs. CONTROL diet (7.0 g/day vs. 4.7 g/day). Participants collected their study foods from Flinders Medical Centre every two weeks, and for those who travelled long distances, at the 4-week time point. At this time, the study researcher interviewed each participant to determine compliance with the study protocol and ease of including study foods into their regular diet. For participants who were finding it difficult to consume the study foods, the study researcher provided verbal motivation for consuming these.

**Table 1:** Composition of the provided study foods

	Energy (kJ)	Protein (g)	Total fat (g)	Saturated fat (g)	Sodium (mg)	EPA (mg)	DHA (mg)	EPA + DHA (mg)	DPA (mg)
FISH: Weeks 1 and 2									
Atlantic salmon-skin off, 145 g^a^	1,056	32.0	13.6	3.2	59	945	1,035	1,980	–
Atlantic salmon-skin off, 145 g^a^	1,056	32.0	13.6	3.2	59	945	1,035	1,980	–
Ocean trout-skin on, 150 g^a^	1,630	28.2	30.3	7.8	41	1,210	2,200	3,430	–
Ocean trout-skin on, 150 g^a^	1,630	28.2	30.3	7.8	41	1,210	2,200	3,430	–
Flavored canned salmon, 95 g^a^	559	13.3	6.8	1.0	271	114	233	347	–
Flavored canned tuna, 95 g^a^	537	17.3	6.3	0.8	326	17	148	164	–
Canned sardines in spring water, 110 g^a^	710	13.7	12.5	3.4	180	750	1,120	1,880	–
Canned pink salmon, 105 g pack^a^	484	18.6	4.9	1.2	280	502	683	1,185	–
									
FISH: Week 3 and 4									
Marinated Atlantic salmon-skin off, 145 g^a^	1,380	27.1	23.5	5.4	312	1,300	1,530	2,830	–
Marinated Atlantic salmon-skin off, 145 g^a^	1,380	27.1	23.5	5.4	312	1,300	1,530	2,830	–
Canned red salmon, 105 g^a^	557	18.4	6.9	1.5	324	494	762	1,256	–
Flavored canned salmon, 95 g^a^	559	13.3	6.8	1.0	271	114	233	347	–
Tuna slices in spring water, 105 g^a^	397	21.7	0.7	0.2	185	29	215	244	–
Lightly seasoned frozen fish fillets, 200 g^a^	1,340	29.2	14.8	2.0	260	200	500	700	–
Canned tuna in spring water, 95 g^a^	227	12.1	0.5	0.3	139	0.2	20.0	147	–
									
MEAT: Weeks 1 and 2									
Beef scotch fillet, lean, raw, 125 g^b^	758.75	29	9.375	2.875	68.75	30	5	35	33.75
Beef scotch fillet, lean, raw, 125 g^b^	758.75	29	9.375	2.875	68.75	30	5	35	33.75
Frozen veal schnitzel, 140 g^c^	1,289	18.06	14.56	4.48	882				
Lamb rump, lean, raw, 125 g^b^	677.5	28.125	5.375	1.75	81.25	23.8	13	36.8	46.3
Beef scotch fillet, lean, raw, 125 g^b^	758.75	29	9.375	2.875	68.75	30	5	35	33.75
Ham deli meat, 50 g^c^	184	7.9	1.5	0.3	293				
Ham deli meat, 50 g^c^	184	7.9	1.5	0.3	293				
									
MEAT: Week 3 and 4									
Beef mince, 10% fat, 150 g^b^	1,112	30.15	16.2	5.85	96	141	31.5	172.5	217.5
Beef mince, 10% fat, 150 g^b^	1,112	30.15	16.2	5.85	96	141	31.5	172.5	217.5
Lamb rump, lean, raw, 125 g^b^	677.5	28.125	5.375	1.75	81.25	23.8	13	36.8	46.3
Beef scotch fillet, lean, raw, 125 g^b^	758.75	29	9.375	2.875	68.75	30	5	35	33.75
Pork loin chop, 140 g^b^	440	22.2	1.7	0.6	66	0	6	6	9
Frozen beef satay convenience meal, 375 g^c^	2,378	25.9	20.6	8.6	675				
Beef scotch fillet, lean, raw, 125 g^b^	758.75	29	9.375	2.875	68.75	30	5	35	33.75
Pork loin chop, 140 g^b^	440	22.2	1.7	0.6	66	0	6	6	9

a Analyses were conducted by AsureQuality Limited (Auckland New Zealand). All samples submitted for analyses were composite samples from a minimum of three production dates. Samples were homogenized with stainless steel cutters, packed in plastic bags protected from light and frozen before being shipped to the laboratory. All samples were analyzed in duplicates. Reagent blank (same procedure but omitting the sample), spikes and standard reference materials were prepared as per the laboratory's standard operating procedure. The laboratory contracted to undertake the analyses was accredited and complied to the requirements for the competence of testing and calibration laboratories ISO/IEC 17025:2005.

b Nutrient profiles were obtained from AUSNUT 2007

c nutrient information panel of actual products.

### Subjects

A total of 126 community dwelling men and women ≥64 years of age expressed interest in the study following newspaper advertisements, flyers and verbal invitations. Ninety-two were eligible for screening; however eight did not attend. Inclusion criteria were: BMI ≥18.5 kg/m^2^, usual consumption of ≤1 serving of fish/seafood per week, willing to consume eight servings of fish or red meat per fortnight, able to provide written informed consent and attend assessment visits at Flinders Medical Centre. Exclusion criteria were: allergies to fish/seafood, vegetarian, intake of lipid-lowering medications; intake of lipid-lowering supplements (e.g. psyllium, fish oil capsules, soy lecithin, phytoestrogens or to cease 3 weeks prior to study commencement), use of anti-inflammatory medications on a regular basis or if experiencing an acute episode within 1 week of the screening visit, presence of diabetes, liver, kidney, thyroid diseases (unless controlled and stable on replacement medication), presence of other endocrine disorders from self-reported medical history, weight loss or gain of >10% body weight in the prior 6 months, or clinically diagnosed depression or dementia. Of the 84 respondents who attended the clinic visit, all were eligible for the RCT, however one participant withdrew before randomization, leaving 83 to be randomized. The Southern Adelaide Clinical Research Ethics Committee granted ethical approval. The clinical trial was registered with the Australian New Zealand Clinical Trials Registry, trial number 336393. Written informed consent was obtained from all subjects.

### Clinic measurements

All assessments were taken at baseline and at 8-weeks. Body weight and height were measured to 0.1 kg and 0.1 cm, respectively, while wearing light clothing and without shoes, on a digital height and weight wireless measuring station (SECA, model 284, Germany). Blood pressure was measured using a hospital grade non-invasive automated sphygmomanometer (Criticare Systems Inc., USA). After 5 min of rest, blood pressure was taken three times and the average reading was used.

### Laboratory measurements

At baseline and at 8-weeks, participants arrived at the clinic following a 12-h overnight fast in which only water was allowed to be consumed. Nursing staff confirmed the fast before taking 10 ml of blood by asking them their last mealtime from the prior evening. After 30 min of clotting, whole blood was centrifuged at 3,500 RPM, 24°C for 10 min (Beckman GS-6R). Serum was separated, aliquoted, and stored at −80°C until all participants completed the study. Aliquots were then thawed, and total cholesterol, HDL-C, triacylglycerol, and high-sensitive CRP (hs-CRP) (limit of detection ≤0.3 mg/L) were analyzed enzymatically (Siemens Advia 2,400 Chemistry System). LDL cholesterol was calculated using the Friedewald Equation (39). Inflammatory cytokines (IL-6, IL-1β and TNF-α) were measured using commercial cytokine enzyme-linked immunosorbent assays (ELISA) (BD Systems, San Diego, CA). The limits of detection for IL-6, IL-1β and TNF-α were all <5 pg/ml. The inter-assay and intra-assay CV for IL-6 were 6–8 and 2–3%, for IL-1β were 7–10 and 2–4% and for TNF-α were 3–5 and 1–3%, respectively.

### Compliance

#### Three-day weighed food records

Dietary intakes were measured using 3-day weighed food records (any 2 week days and any 1 weekend day) and were completed during week 1 and week 7 of the study to assess study compliance. All participants were instructed on how to fill out their food records at the baseline visit and were provided a set of digital food scales (Kenwood DS607 Digital Food Scales, Australia).

#### Fatty acids

Compliance was assessed using measurement of fatty acids in erythrocytes and plasma phospholipids (DHA, DPA, EPA, total n-3 fatty acids, total n-6 fatty acids, and total saturated fatty acids) collected into heparinized tubes.

##### Lipid extraction from plasma phospholipids

Plasma was separated from erythrocytes by centrifugation (5 min at 1,000 *g*) and 1 ml plasma was added to 0.5 ml saline, 2 ml of methanol and 4 ml of chloroform. The samples were centrifuged and the lower chloroform phase (containing lipids) was transferred to a 20 ml scintillation vial and evaporated to dryness by a vacuum concentrator. 150 ml of chloroform: methanol (9:1) was added to the vial in preparation for thin layer chromatography (TLC).

##### Lipid extraction from erythrocytes

Erythrocytes were rinsed free of plasma three times in isotonic saline and 1 ml packed cells were mixed with 0.5 ml saline, 2 ml of isopropanol, and 4 ml of chloroform. The samples were centrifuged and the lower chloroform phase was transferred to a vial and evaporated to dryness. About 150 ml of chloroform: methanol (9:1) was added to the vial in preparation for TLC.

##### Thin Layer Chromatography

The phospholipids from all tissues were isolated using TLC (Silica gel 60 H, Merck, Darmstadt Germany) using a ratio of 3:1 (v/v) of petroleum spirits: acetone. Phospholipid bands were visualized under an ultraviolet lamp and scraped into vials containing 1% H_2_SO_4_ in methanol for trans-esterification for 3 h at 70°C. After cooling, water and *n*-heptane were added to the mixture and the upper layer containing the resulting fatty acid methyl esters (FAME) was transferred into 2 ml gas liquid chromatography (GLC) vials containing anhydrous Na_2_SO_4_.

##### Gas chromatographic analysis of FAME

FAMEs were separated and quantified using a Hewlett-Packard 6,890 gas chromatograph equipped with a 50 m capillary column (0.33 mm ID) coated with BPX-70 (0.25 m film thickness SGE Pty Ltd Victoria Australia). The injector temperature was set at 250°C and the detector (flame ionization) temperature at 300°C. The initial oven temperature was 140°C and was programmed to rise to 220°C at 5°C per minute. Helium was used as the carrier gas at a velocity of 35 cm per second. Individual fatty acids were identified by comparison with known fatty acid methyl ester standards (Nuchek Prep Inc., Elysian, USA) and expressed as a percentage of total fatty acids (weight percentage) (40).

### Sample size

The sample size was based on previous studies that showed a 19% (0.4 mg/L) reduction in CRP following 360 mg/day EPA + DHA as fish oil for 3 weeks in hyperlipidemic patients (*n*=15, ∼55 years) (41); a 13% (0.19 mg/L) reduction in CRP following 2 × 150 g/week of salmon for 6 months (1,400 mg/day total omega-3; *n*=54, ∼54 years) (29); and an 8% (0.13 mg/L) reduction in CRP following 2×150 g/week cod (257 mg/day total omega-3 for 6 months; *n*=51, ∼57 years) (29). The calculated sample sizes required from these respective studies were 20, 8 and 17. As the target study population was older, free of disease, and may have a slightly higher inflammatory status at baseline, we anticipated a larger sample size would be required to find a similar significant change in CRP from the FISH diet. Therefore, the current study aimed to recruit 90 subjects in order to have 40 subjects complete the 8-week study in each group, with sufficient power to detect a CRP change of 13% (0.24 mg/L) for those allocated to the FISH diet. An independent statistician using ralloc.ado version 3.6.1 in Stata version 11.1 created the randomization schedule. The randomization was stratified by screening CRP concentrations (<3 mg/L vs. ≥3 mg/L) in order to determine whether diet affected CRP differently between those with different baseline CRP concentrations. Block sizes of 2, 4 and 6 were used and were chosen at random; treatment allocations were randomly permuted and balanced within blocks.

### Statistical analyses

Statistical analyses were performed using SPSS 19 for Windows (SPSS, Inc., Chicago, IL, USA). Frequencies and descriptives of the study population are reported as mean (SD), or between groups as mean (SEM). Prior to hypothesis testing, data were examined for normality. Distribution was normal except for CRP, IL-1 and IL-6, which were normalized using natural logarithmic transformation with the exponentiated (geometric) means (95% confidence intervals) presented. Primary and secondary outcomes at 8-weeks were tested using ANCOVA (univariate analysis of variance, adjusting for baseline values). As a secondary analysis, the absolute change in CRP was assessed with CRP strata as a fixed factor (group×CRP stratification interaction), without adjustment for baseline CRP. Adjustment for multiple comparisons was made using the Sidak correction. The alpha level of significance was *p*<0.05.

## Results

### Baseline characteristics

A total of 83 participants started the study; however one participant withdrew from the FISH diet (too ill to continue, aged 81 years) and two withdrew from the CONTROL diet (*n*=1 did not turn up for final visit despite several phone calls and emails, aged 72 years; and *n*=1 chose to continue with Zocor medication 2 weeks into the study, aged 66 years), leaving 80 participants to complete the study. The mean (SD) age of completed participants was 69.6 (5.8) years (range 64–85 years) and 51% were female. Mean (95% CI) baseline CRP concentrations of the completed participants in the low and high CRP strata are shown in Supplementary file. There were no baseline differences in CRP strata concentrations between those allocated to the FISH diet and those allocated to the CONTROL diet. Characteristics of the study participants are represented in Table 2.

**Table 2:** Baseline and 8-week measurements amongst the study population

	Baseline				Week 8			
		
	Control^a^		Fish^a^		Control^a^		Fish^a^	
				
	*n*		*n*		*n*		*n*	
Weight (kg)	37	73.8±2.1	43	75.7±2.2	37	73.6±2.0	43	75.8±2.1
BMI (kg/m^2^)	37	26.4±0.6	43	26.5±0.6	37	26.3±0.6	43	26.5±0.6
Systolic blood pressure (mmHg)	37	126±2	43	126±2	37	126±3	43	124±3
Diastolic blood pressure (mmHg)	37	67±1	43	69±1	37	66±1	43	68±2
CRP (mg/L)^b^	37	0.8 (0.5, 1.3)	43	1.17 (0.8, 1.8)	37	0.8 (0.5, 1.3)	43	1.0 (0.7, 1.5)
IL-1β (pg/ml)^b^,^c^	15	6.9 (4.7, 10.1)	18	8.11 (5.3, 12.4)	15	7.7 (6.2, 9.4)	18	7.3 (6.0, 8.8)
IL-6 (pg/ml)^b^,^c^	10	7.9 (5.4, 11.4)	11	7.21 (5.4, 9.7)	10	7.5 (5.9, 9.6)	11	7.7 ( 5.2, 11.4)
Total cholesterol (mmol/L)	37	5.5±0.2	43	5.5±0.1	37	5.4±0.2	43	5.6±0.1
HDL-C (mmol/L)	37	1.6±0.1	43	1.7±0.1	37	1.7±0.1	43	1.8±0.1
LDL-C (mmol/L)	37	3.3±0.1	43	3.2±0.1	37	3.2±0.1	43	3.4±0.1
Triacylglycerol (mmol/L)	37	1.4±0.1	43	1.1±0.1	37	1.3±0.1	43	1.0±0.1

BMI: body mass index; CRP: C-reactive protein; IL-1β: interlukin-1 beta; IL-6: interleukin 6; HDL-C: high-density lipoprotein cholesterol; LDL-C: low-density lipoprotein cholesterol.

a Data reported as mean±standard error of the mean.

b Data reported as geometric means (95% CI).

c Values only reported if above limit of detection.

### Study compliance

#### Food intake

Table 3 describes the mean gram intake and some nutrient intakes consumed from the study fish and meat, as well as by non-study fish/meat during week 1 and week 7 of the study, in FISH and CONTROL. For those allocated to the FISH diet, no other fish was consumed at either time point. For those allocated to the CONTROL diet, one serving of fish was consumed by one participant in week 1 and week 7 of the study. Supplementary file shows the mean energy and nutrient intakes between groups during week 1 and week 7 of the study. Intakes of VLCN n-3 PUFA was higher for those allocated to the FISH diet at both time points compared to those allocated to the CONTROL diet, whereas the intakes of most other nutrients were generally not different between groups.

**Table 3:** Mean (SD) daily study meat and fish consumed and key nutrient intakes from these foods at week 1 and week 7 of the study

	Gram (g)		Energy (kJ)		Protein (g)		Total fat (g)		Saturated fat (g)		VLCN n-3 PUFA	
						
FISH	Week 1	Week 7	Week 1	Week 7	Week 1	Week 7	Week 1	Week 7	Week 1	Week 7	Week 1	Week 7
Study fish consumed	121 (45)	124 (47)	920 (520)	881 (461)	23 (10)	24 (11)	13 (10)	11 (8)	3.1 (2.7)	2.6 (2.4)	1,752 (1,262)	1,399 (1,244)
Non-study meat consumed^a^	149 (92)	153 (76)	1,102 (775)	1,182 (688)	36 (26)	31 (17)	12 (11)	13 (11)	4.3 (4.7)	5.2 (5.7)	86 (119)	50 (71)
CONTROL												
Study meat consumed	123 (78)	127 (65)	857 (580)	987 (505)	30 (18)	33 (15)	8 (6)	9 (6)	2.8 (2.7)	3.4 (2.6)	62 (62)	80 (60)
Non-study meat consumed^b^	142 (54)	142 (18)	1,074 (504)	1,077 (774)	31 (14)	26 (20)	11 (8)	13 (11)	4.4 (4.1)	4.9 (4.9)	65 (78)	195 (486)

SD: Standard deviation; VLCN: very long chain omega-3 polyunsaturated fatty acids.

a No other fish was consumed, therefore this intake represents intake of meat/chicken/pork at the appropriate time points.

b Only one serving of fish was consumed by one participant during week 1 and week 7 of the study, therefore this intake represents additional intake of meat/chicken/pork at the appropriate time points.

#### Fatty acids

Mean ± SEM percentage VLCN n-3 PUFA in erythrocytes at 8-weeks was higher for those allocated the FISH diet vs. CONTROL diet (10.2±0.2% vs. 8.2±0.3%, *p*<0.001) (Table 4) and this was also true of plasma phospholipids (Supplementary file). Percentage total n-6 fatty acids in plasma phospholipids, but not erythrocytes, was lower in those allocated the FISH diet vs. CONTROL diet at 8-weeks_._

**Table 4:** Mean (±SEM) percentage concentrations of fatty acids measured in erythrocytes at baseline and after 8-weeks consumption of a CONTROL (*n*=37) or FISH (*n*=43) diet

	Group	Baseline (%)	Week 8 (%)
Total saturated fatty acids	Control	44.2±0.2	44.1±0.3
	Fish	44.1±0.2	44.8±0.2
Total n-6 fatty acids	Control	27.0±0.4	26.6±0.4
	Fish	27.2±0.4	25.5±0.3†
Arachidonic acid	Control	12.0±0.3	12.4±0.2
	Fish	12.5±0.2	11.8±0.2*
Total monounsaturated fatty acids	Control	18.4±0.2	17.8±0.2
	Fish	18.2±0.2	17.7±0.2
Total n-3 fatty acids	Control	9.7±0.4	8.4±0.3
	Fish	9.3±0.3	10.4±0.2‡
Alpha linoleic acid	Control	0.147±0.008	0.149±0.007
	Fish	0.126±0.008	0.136±0.008
Linoleic acid	Control	10.3±0.2	9.8±0.2
	Fish	10.2±0.2	9.8±0.2
VLCN n-3 PUFA	Control	9.5±0.4	8.2±0.3
	Fish	9.2±0.3	10.2±0.2‡
EPA	Control	1.5±0.1	1.1±0.3
	Fish	1.3±0.1	1.7±0.1‡
DPA	Control	3.0±0.1	2.8±0.1
	Fish	2.8±0.1	2.8±0.1
DHA	Control	5.0±0.2	4.4±0.2
	Fish	5.0±0.2	5.7±0.1‡

SEM: standard error of the mean; VLCN n-3 PUFA: very long chain omega-3 polyunsaturated fatty acids; EPA: eicosapentaenoic acid; DPA: docosapentaenoic acid; DHA: docosahexanoic acid.

* *p*<0.05

† *p*<0.01

‡ *p*<0.001 between groups (analyzed using univariate analysis of variance, adjusting for baseline values).

### Primary outcomes: inflammatory cytokines

**Fig. 1:**
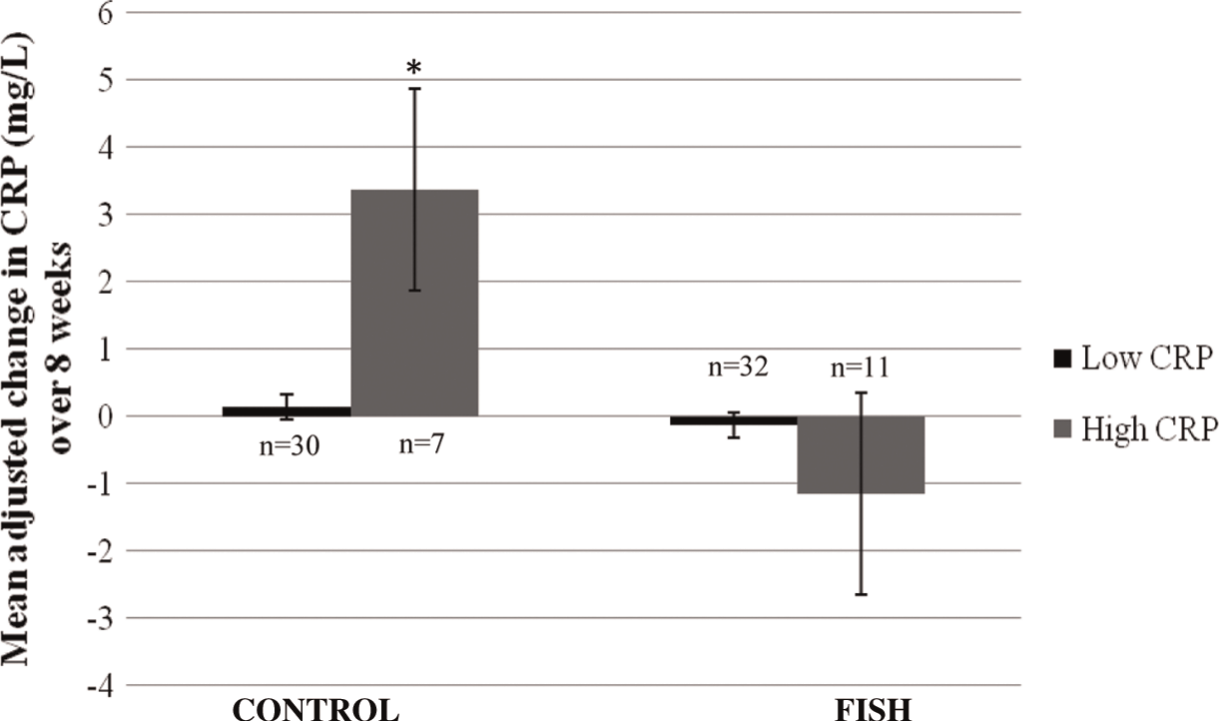
Mean adjusted changes in CRP over 8-weeks between CONTROL and FISH Group×CRP strata interaction (*p*=0.017) using the Sidak test for multiple comparisons. *p=0.046 between high and low CRP in CONTROL.

The mean values of CRP between groups are reported in Table 2. There was no difference in 8-week CRP concentrations between groups. In the secondary analysis assessing the change in CRP over time, there was a diet effect according to CRP strata (*p*=0.017): in CONTROL, those with high CRP had a larger increase in CRP over the study (*n*=7, Δ = + 3.37 (3.24) mg/L) compared to those with low CRP (*n*=30, Δ = + 0.14 (0.23) mg/L, *p*=0.046, Fig. 1). The change in CRP in the FISH group was not different between those with low (*n*=32, Δ = − 0.13 (0.18) mg/L) or high CRP (*n*=11, Δ = − 1.15 (1.5) mg/L, *p*=0.27).

All of the TNF-α serum samples were below the limits of detection (<5 pg/ml) and were therefore unable to be included in the analyses. Mean concentrations of IL-1β and IL-6 that were detectable in serum are reported in Table 2. Diet allocation had no effect on IL-1β or IL-6 at 8-weeks.

### Secondary outcomes: lipids, blood pressure

There was no effect of diet allocation on blood pressure or lipids (Table 2).

## Discussion

This project was the first to be conducted among a group of healthy older adults in Australia, to assess whether 8-weeks consumption of four servings/week mixed fish reduces inflammatory cytokines, compared to a typical Australian diet that is lower in fish and higher in red meat. The current study identified that compared to a low fish diet, consumption of four servings/week fish did not affect concentrations of cytokines overall, nor have any effect on the secondary outcomes, blood pressure and lipids. In a secondary analysis assessing the change in CRP over time, there was a diet effect according to CRP strata; those with CRP concentrations ≥3 mg/L in the control group had larger increases in CRP over the study compared to those with CRP concentrations <3 mg/L; fish had no effect on the change in CRP in participants with CRP ≥3 or <3 mg/L.

Compliance with the intervention was appropriate as we found an increase in VLCN n-3 PUFA in erythrocytes in the fish group, and a decrease in the control group. Consumption of fish and meat, assessed by the 3-day weighed food records in weeks 1 and 7, was also satisfactory with high intakes of VLCN n-3 PUFA in the fish group. The amount of non-study meat that was consumed at both time points was similar between groups, and it was evident that the control group adhered to the study protocol as, except for one participant, no fish was consumed. We did not find any changes in percent n-6 fatty acids or DPA in erythrocytes (or plasma phospholipids) in the control group, therefore we can speculate that intake of red meat during the study period was comparable to usual intake.

In the current study, the increased consumption of fish did not decrease serum cytokines. The mechanisms relating long chain fatty acids and inflammation are emerging and are the result of the increased incorporation of VLCN n-3 PUFA into plasma phospholipids (42). Arachidonic acid (ARA, an omega 6 PUFA) is usually the major substrate for eicosanoid synthesis; eicosanoids are the most important mediators and regulators of inflammation (43). The incorporation of the VLCN n-3 PUFA (EPA and DHA) occurs at the expense of ARA. Thus, the normal pro-inflammatory response that would occur in response to ARA and its derivatives (e.g. prostaglandins, thromboxanes and leukotrienes) is reduced. The increased incorporation of VLCN n-3 PUFA and decreased ARA leads to a dampening of the pro-inflammatory state, and toward an anti-inflammatory state. Therefore, the fact that the increased intake of fish did not impact measures of inflammation indicates that either the dose of VLCN n-3 PUFA may have been too low, or that initial inflammatory status was not high enough to identify a notable effect. However, the sample size in which concentrations of IL-1β (*n*=33) and IL-6 (*n*=21) was detected was small and therefore the low statistical power along with large variation in cytokine concentrations, would partly explain our result. Cell culture studies and some animal feeding studies have shown that cytokine inhibition by omega-3 is mediated by inactivation of NFκB, a principle transcription factor (44). It would have been of interest to assess gene expression in this study and assess how fatty acids might affect activation of NFκB in our healthy older adult sample.

Several other RCTs in older adults have assessed fish consumption on inflammatory cytokines (25–31); however, these studies were conducted in those who were medically unwell or were in conjunction with other therapies. The increase in VLCN n-3 PUFA following the fish diet in our study was slightly lower compared to other intervention studies utilizing fish; however, the prescribed daily VLCN n-3 PUFA dose from fish in our study was also generally lower (∼800 mg/day vs. >1.2 g/day). Although our intention was to use healthy individuals, the specific use of older adults was novel and we anticipated a higher inflammatory status as a result of the aging process (irrespective of this group having no risk factors for CVD). Mean CRP values at baseline were comparable to those previous studies that assessed fish and inflammation, but the effect of increasing VLCN n-3 PUFA appears most effective with higher levels of inflammation. This conclusion was also indicated in a previous review, such that patients with an inflammatory condition might be more sensitive to the immunomodulatory effects of VLCN n-3 PUFA compared to healthy subjects (45).

We used CRP as the primary outcome measure as it has been consistently found to be an independent predictor of CVD (46). The use of an hs-CRP assay also enables low levels of CRP to be detected in peripheral blood, indicating low-grade inflammation (47). We acknowledge that because CRP is an acute-phase reactant some intra-individual variability may occur in testing, and low-grade inflammation can produce minor elevations of CRP. Although we attempted to have all participants’ blood taken while they were free of any symptomatic illness, the low level of CRP at baseline suggests that amongst healthy older adults, the benefits of increased consumption does not provide an anti-inflammatory benefit. It would be of interest to assess the effects of increased fish consumption in a larger group of healthy adults with CRP >3 mg/L, and to determine whether a clinically relevant reduction in CRP occurs.

The effect of VLCN n-3 PUFA on blood pressure has been described; however, the positive effect of high dose VLCN n-3 PUFA supplementation on blood pressure is small (48). For example, in a meta-regression of 36 RCTs, VLCN n-3 PUFA supplementation decreased systolic and diastolic blood pressure in those >45 years by −2.72 and −2.32 mmHg, respectively; greater than what was observed for those ≤45 years (48). However, the median intake of VLCN n-3 PUFA (3.7 g/day) was far higher than that provided by fish in the current study, and would not be a usual intake of VLCN n-3 PUFA when supplements are not consumed. The effect of increased consumption of fish on blood pressure in older adults has not been specifically evaluated. Therefore, this outcome provided another novel component to our study. In our healthy group of older adults, we did not find an effect of increased fish intake on blood pressure. The beneficial effect of fish on blood pressure is thought to be through the increased synthesis of nitric oxide, a potent vasodilator, as well as the reduced production of the vasoconstrictor thromboxane A2, thus decreasing platelet aggregation and improving arterial compliance (49). As the meta-regression by Geleijnse et al. indicated that a greater effect on blood pressure was observed in hypertensives rather than normotensives (48), it is possible that we might have observed an effect on blood pressure if our study population also had elevated blood pressure. Nevertheless, further dose-response intervention studies assessing the relative effects of higher VLCN n-3 PUFA intakes on blood pressure in older adults are required, as well as the effect of increased fish intake in older adults with hypertension would be of value.

In our secondary analysis, among the small sample of adults with high baseline CRP concentrations, an increase in CRP was evident following the control diet, compared to those with low CRP on the control diet. This was a surprising result and it appears this type of diet could be unfavorable in this small group of healthy older adults, at least in the short-term. One study in adult women previously indicated that a higher intake of meat was associated with a higher level of CRP (50); whereas another study reported a diet high in red meat did not increase markers of oxidative stress and inflammation in men and women (51). These findings lend for further studies to assess the potential effects of CRP following a low fish, higher red meat diet, in healthy older adults with high CRP.

For Australian adults to lower their risk for CHD, it is recommended that 500 mg/day of VLCN n-3 PUFA is consumed (two or three servings (150 g serve) of oily fish per week), and for patients with documented CHD is 1,000 mg/day (10). Therefore, the intention to supply the fish in this study at an amount nearly twice the current recommendations was to assist in compliance for a relatively short time-frame, as consumption of fish in this amount is not usual (52, 53). Our results demonstrated that short-term consumption of a high fish diet is feasible. However, a higher fish intake in older adults who do not display cardiovascular and metabolic co-morbidities are unlikely to benefit from a diet higher in fish in an effort to further reduce CVD biomarkers, and thus, does not provide evidence that our results can be generalized into a public health recommendation for fish. We acknowledge that consumption of this amount of fish in the longer term is unlikely to be sustainable and other sources such as a fish oil supplement may be appropriate, if indeed, future studies identified a benefit from further reduction of already desirable cardiovascular biomarkers amongst older adults.

The study duration of 8-weeks was chosen as this time-frame is sufficient to alter the membrane composition and incorporation of omega-3 fatty acids in cells and tissues (54); and changes in IL-6 and TNF-α, which are mediators of CRP, can be noted within 6-weeks following VLCN n-3 PUFA in doses slightly higher (27) and much higher (55, 56) than that used in the current study. Provision of red meat in the control diet was based on the recommended 1–1.5 daily servings of ‘meat, fish, poultry, eggs, nuts and legumes’ from the Australian Guide to Healthy Eating for men and women >60 years (57) and that intake of red meat is generally lower than intake of poultry. We provided four servings of red meat per week, equivalent to ∼0.5 serving/day red meat, or 68 g/day. This intake of meat was intermediate compared to consumption data in previous Australian studies (52, 53). In our study, we did not assess food intake prior to study commencement, therefore it is unclear whether the meat we provided is a usual intake for this group. We speculated that randomization would account for any baseline differences and the primary purpose of the dietary assessment was for compliance purposes. Another measure of dietary intake at baseline would have increased the burden for participants. We found good overall compliance with the study foods; however we did not specify which 3 days food was to be weighed nor on which days the study foods were to be consumed. This presents a further limitation to the study. Finally, we did not use high-sensitive ELISAs for IL-1, IL-6 and TNF-α, resulting in few samples with detectable concentrations. High-sensitive ELISAs would have enabled us to identify detectable cytokine concentrations, and therefore would have assisted in determining whether a higher fish diet affects a composite of cytokine concentrations.

## Conclusions

Eight weeks consumption of four servings of fish per week did not affect serum cytokine concentrations, lipids or blood pressure compared to a diet low in fish. In healthy older adults with low inflammatory burden, our results do not support that short-term consumption of mixed fish has a beneficial effect on selected cardiovascular biomarkers. Although a small sample size, the secondary analysis indicates that a diet more consistent with Australian dietary guidelines that is higher in meat and lower in fish, might increase CRP among older adults with CRP ≥3 mg/L. Further, larger studies utilizing the same high fish intervention are required to substantiate this finding in healthy older adults with high CRP.
